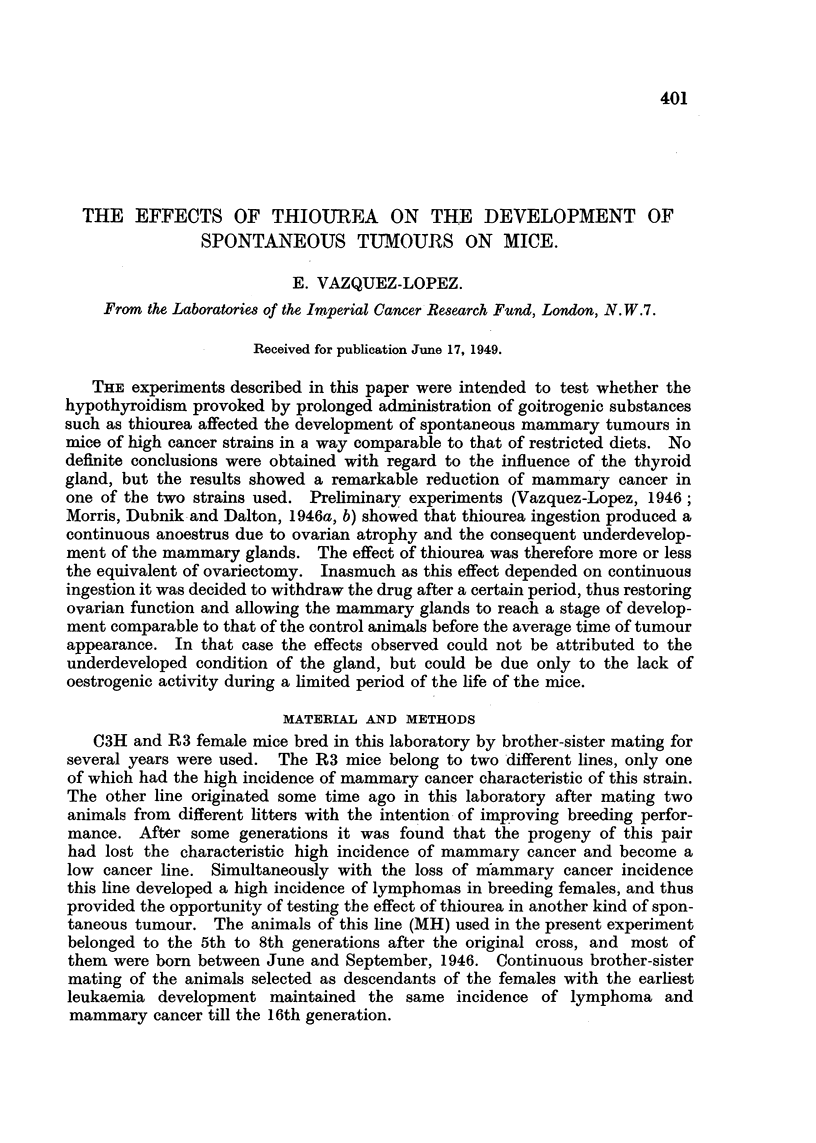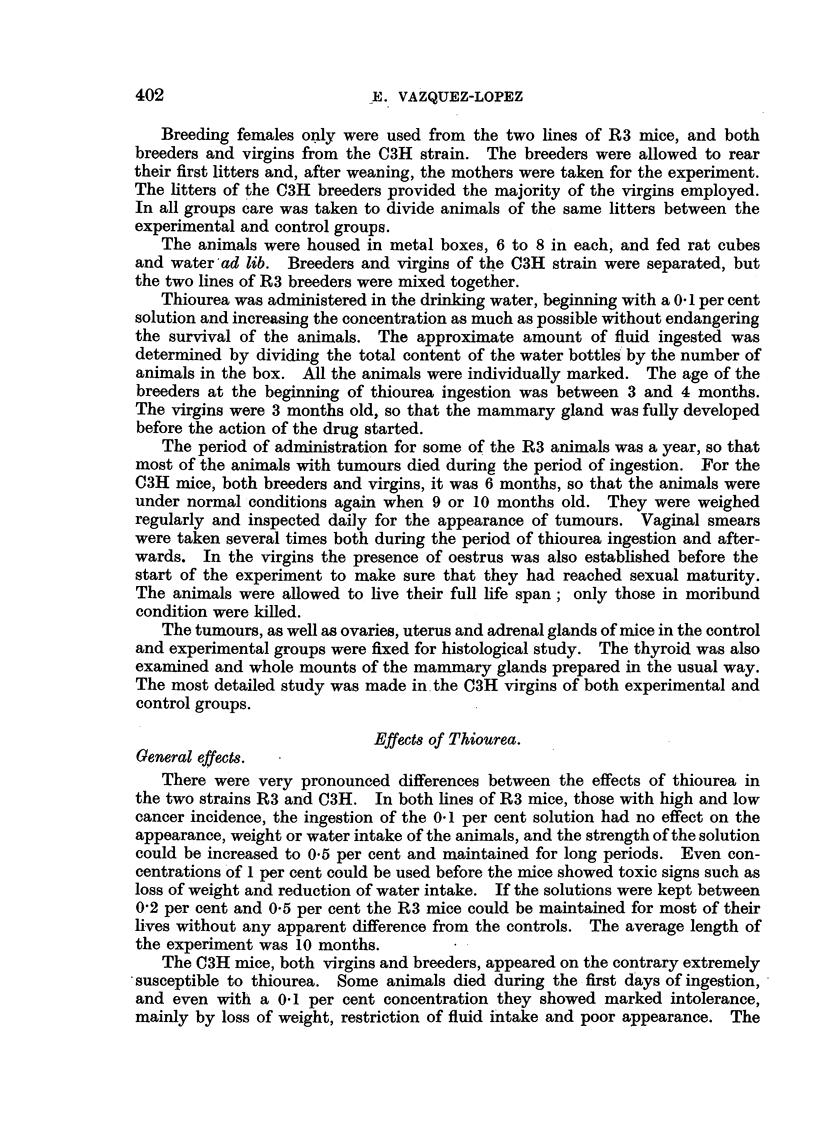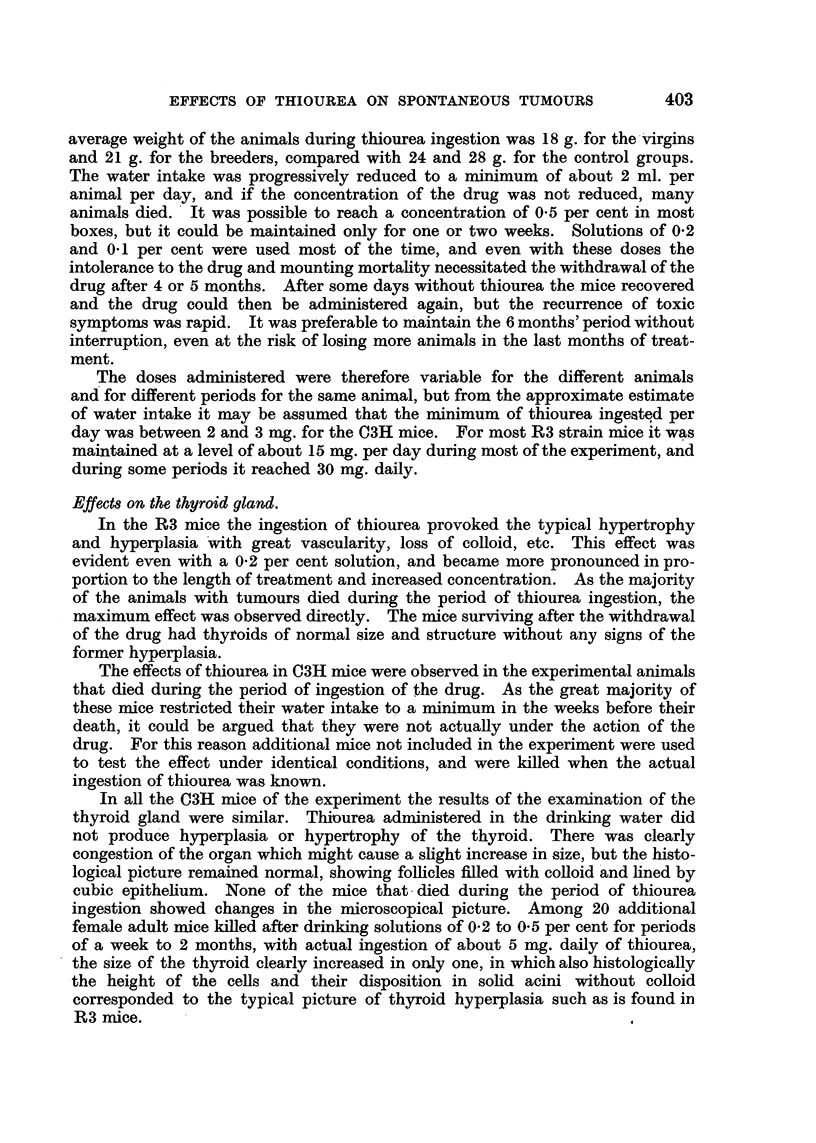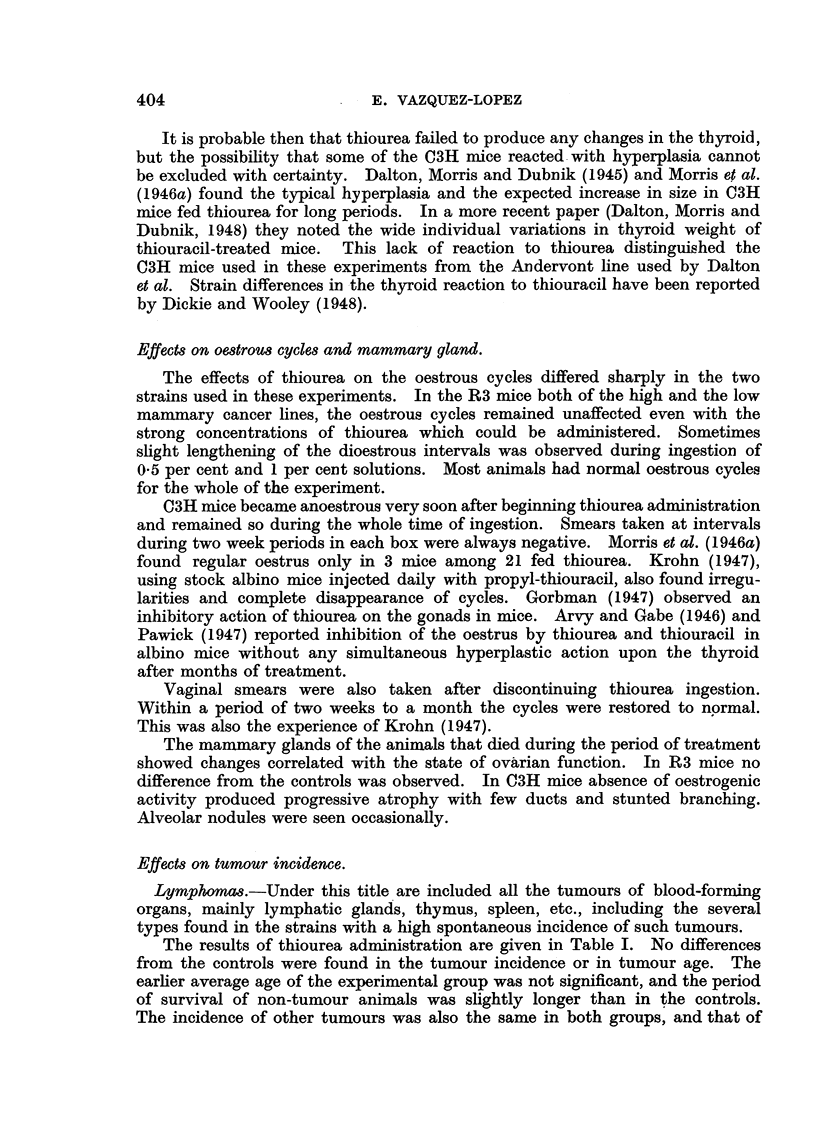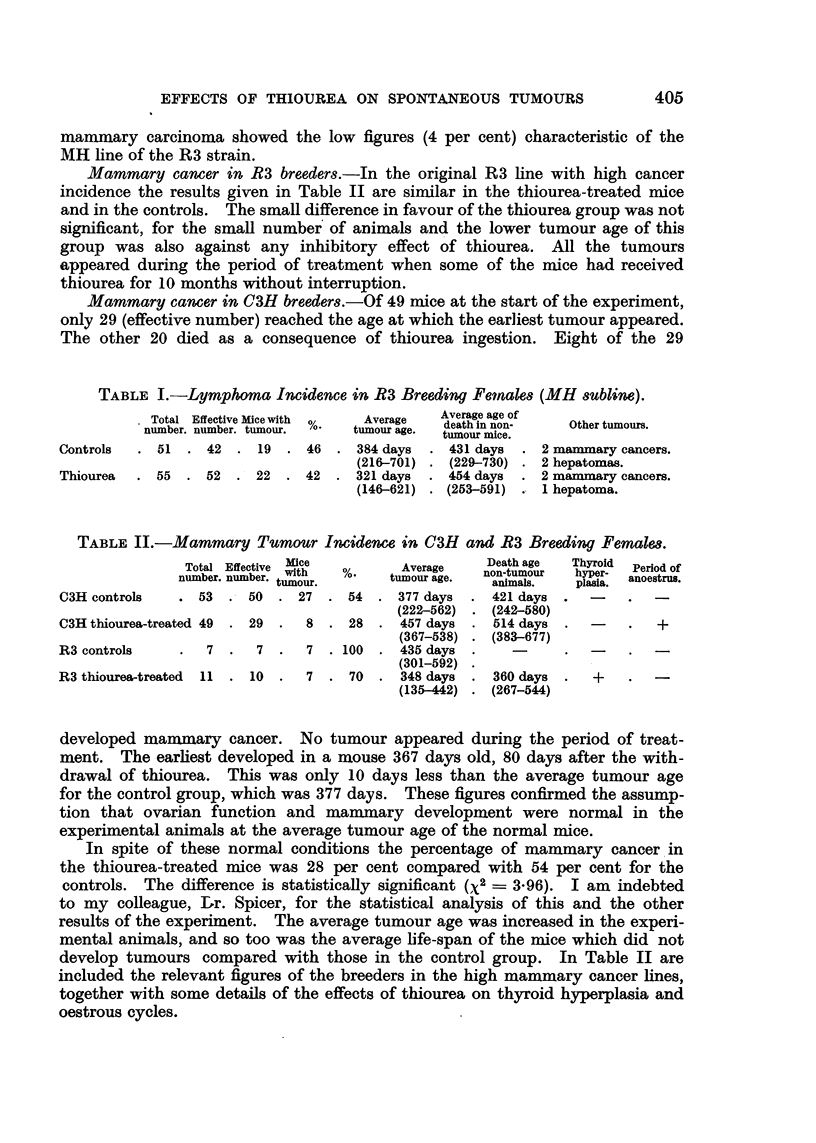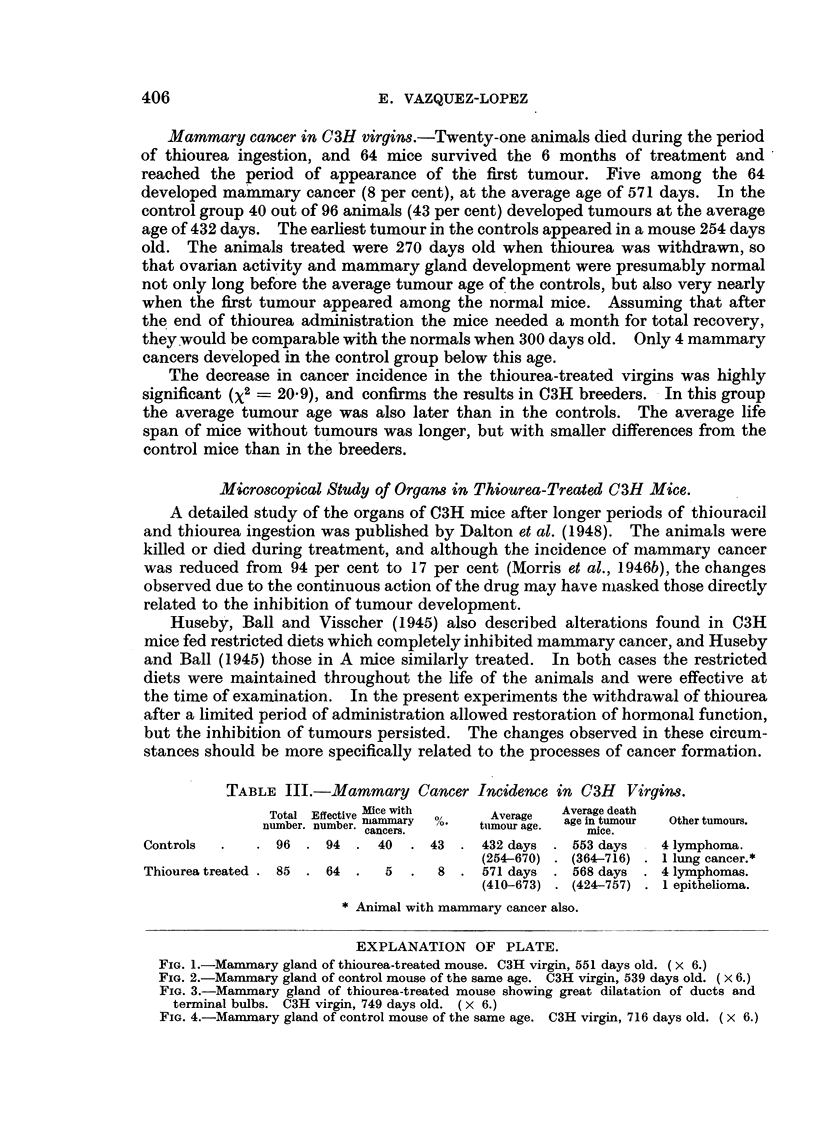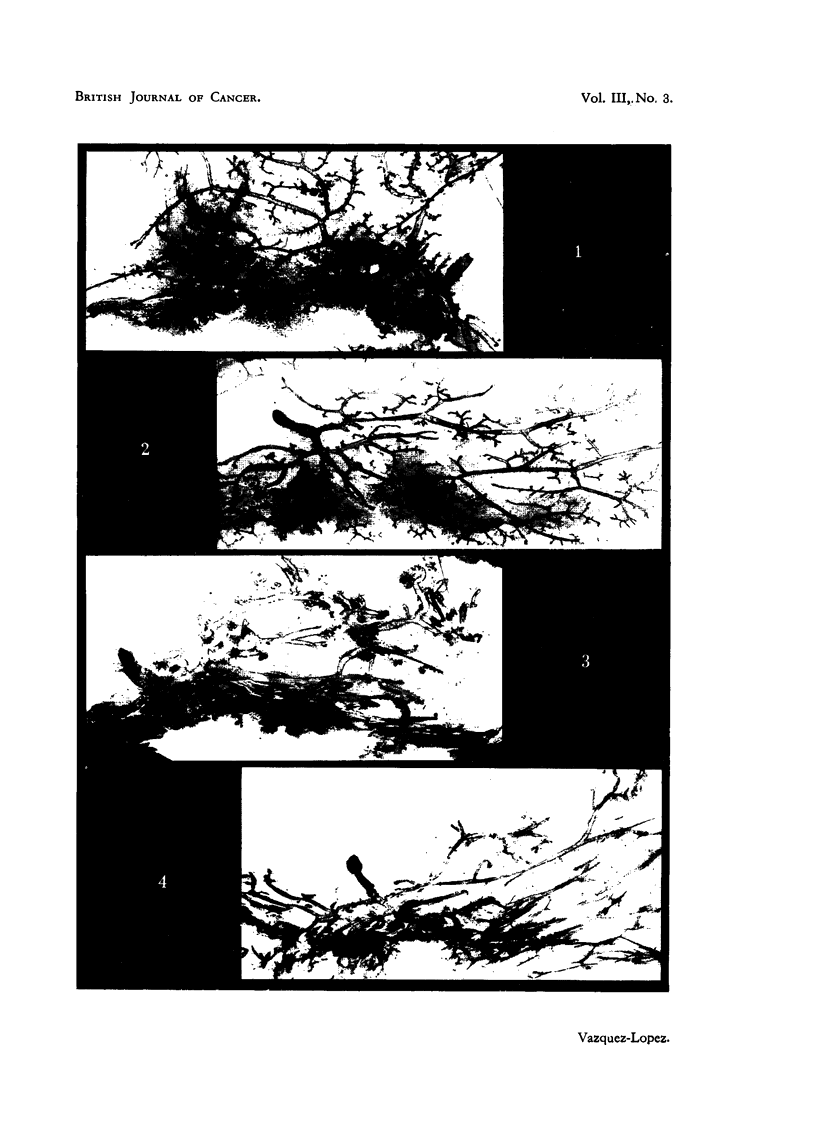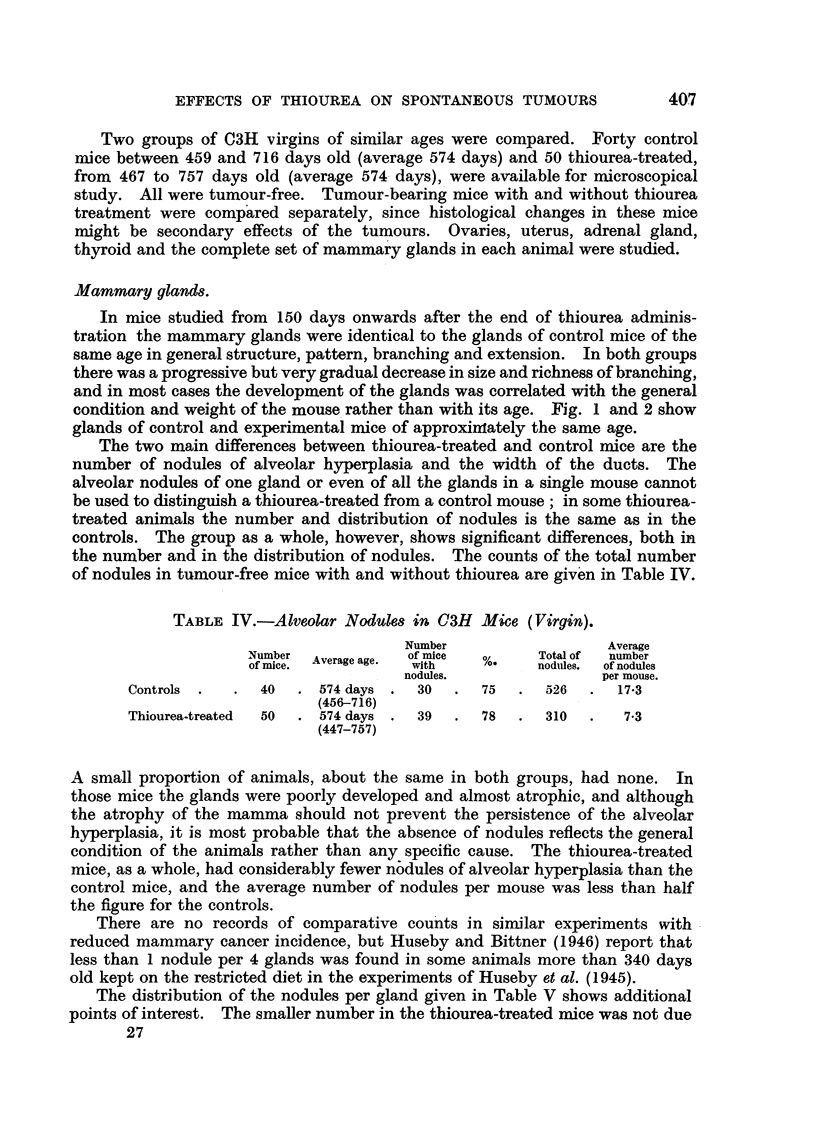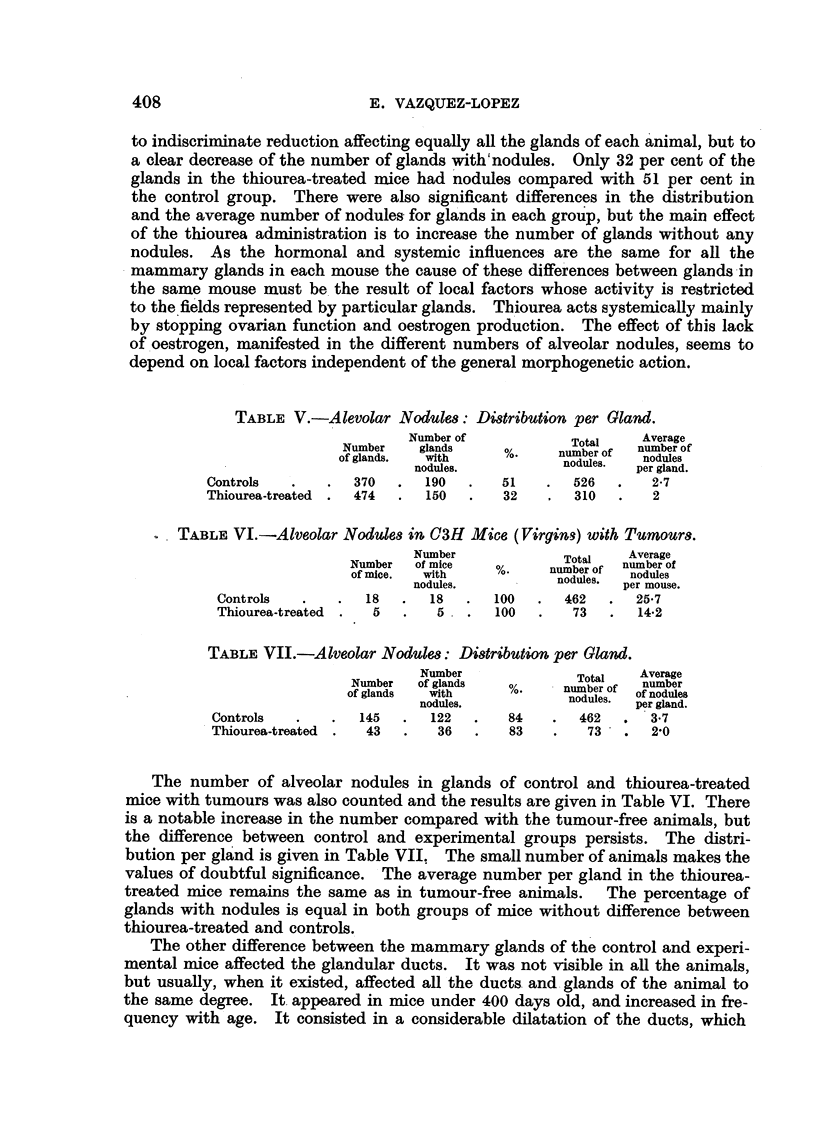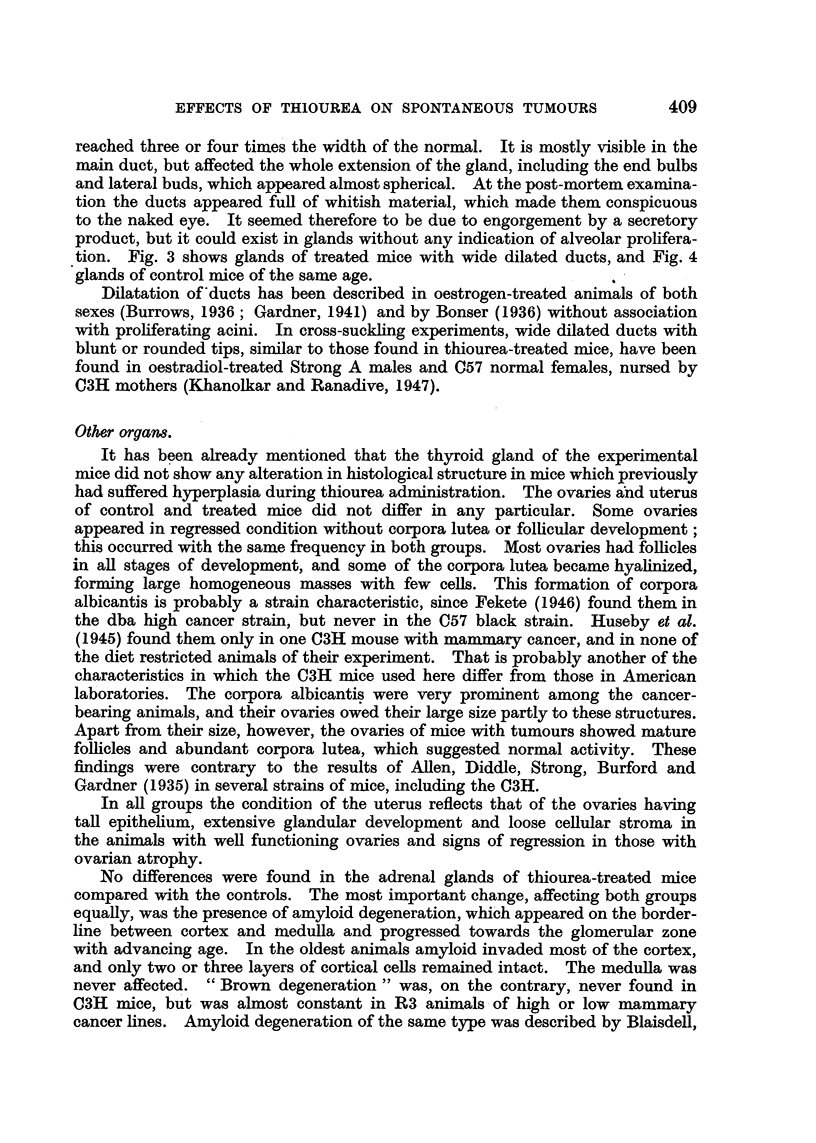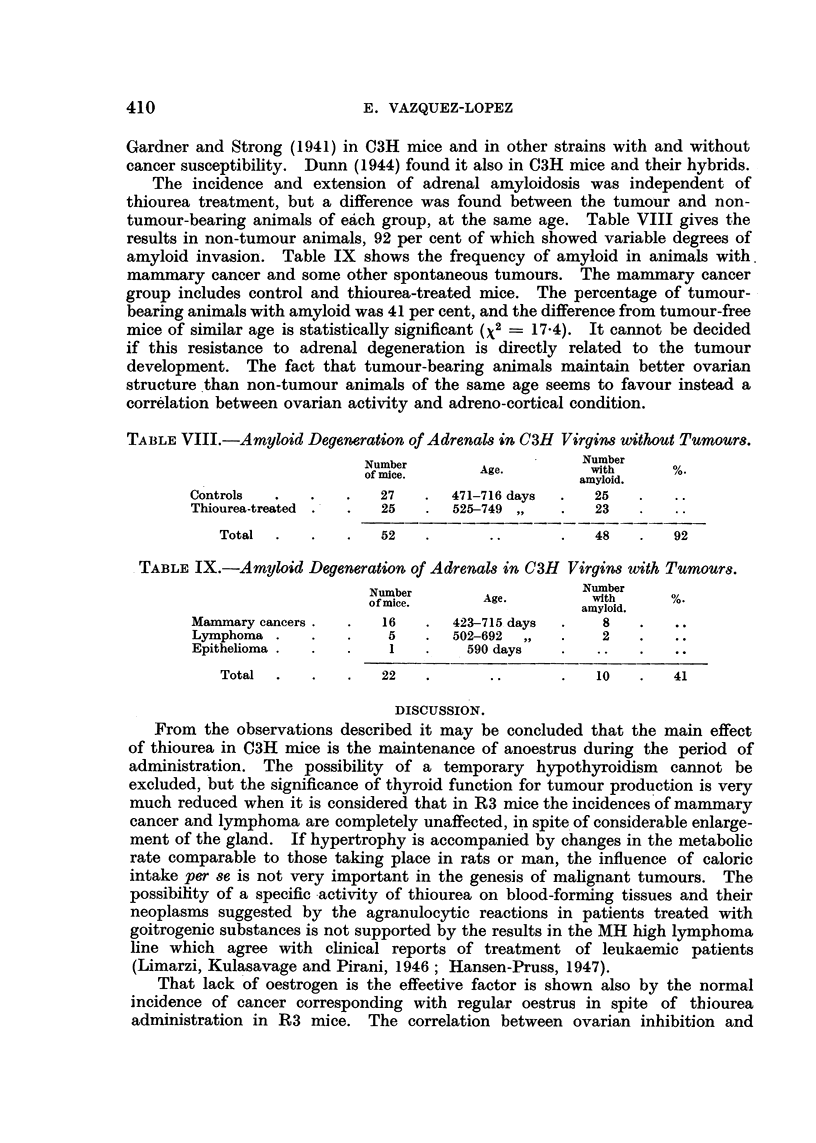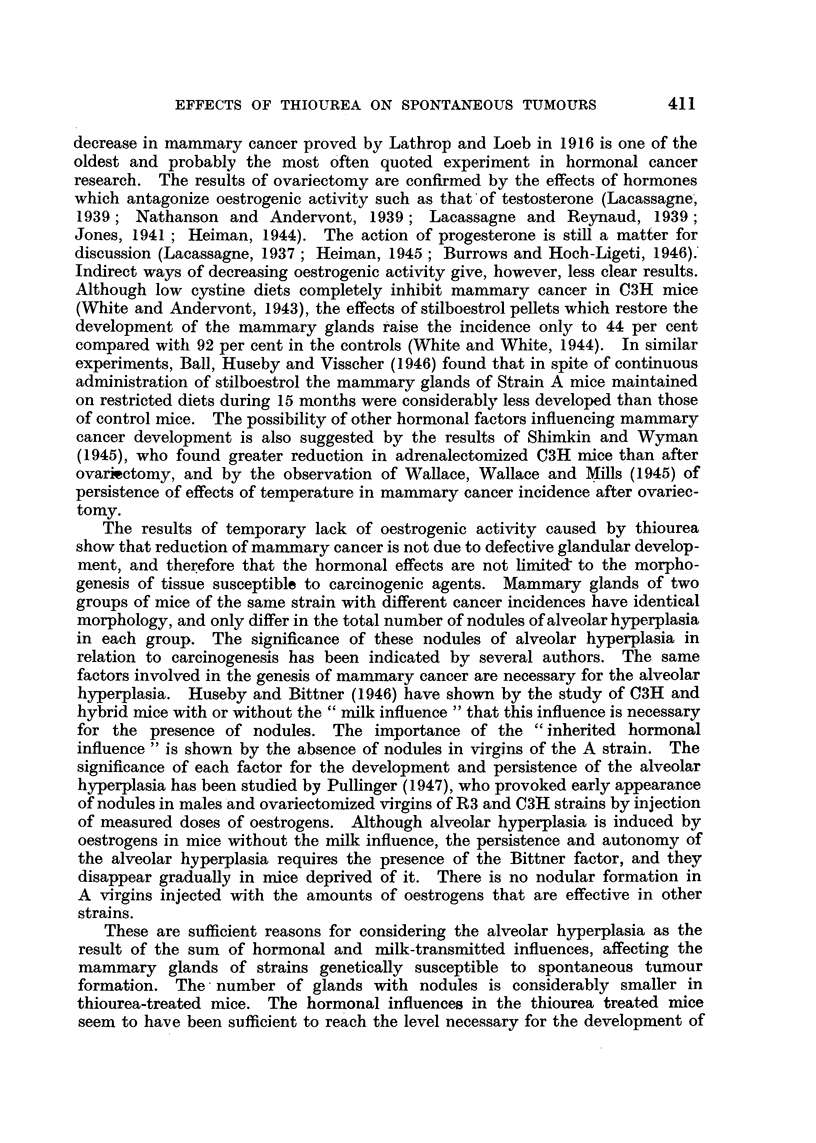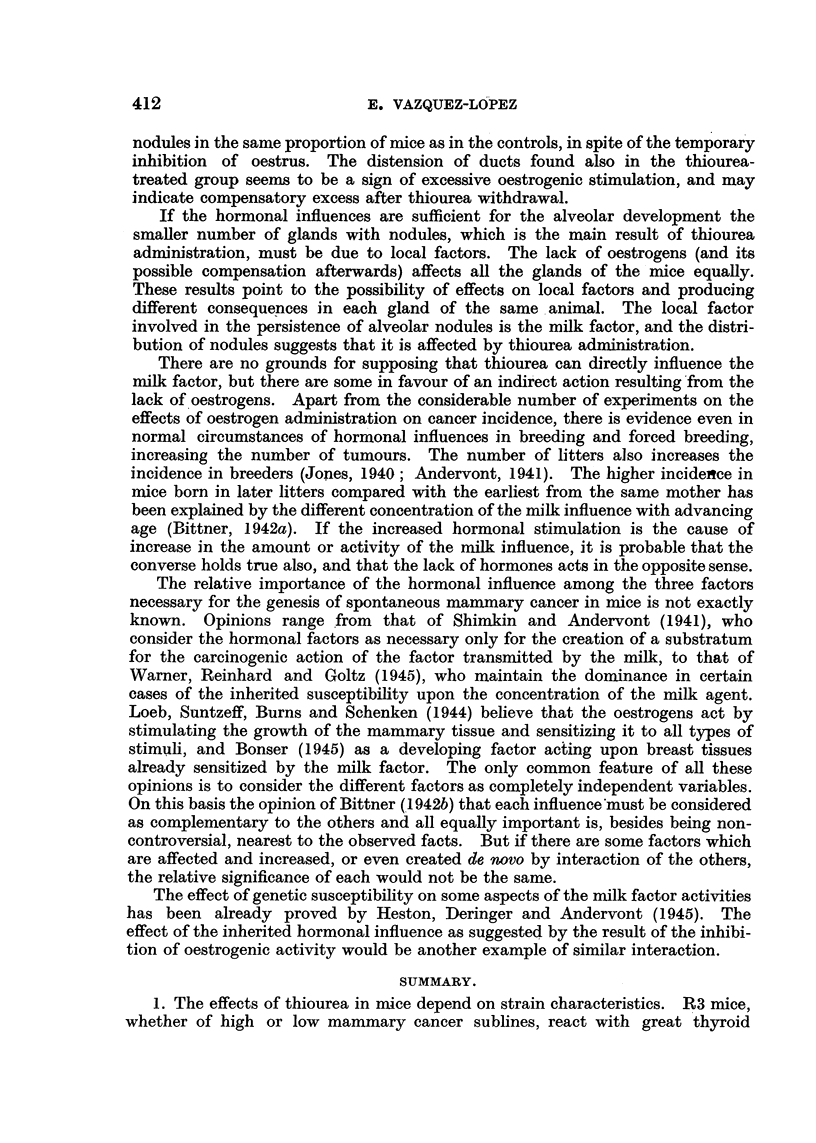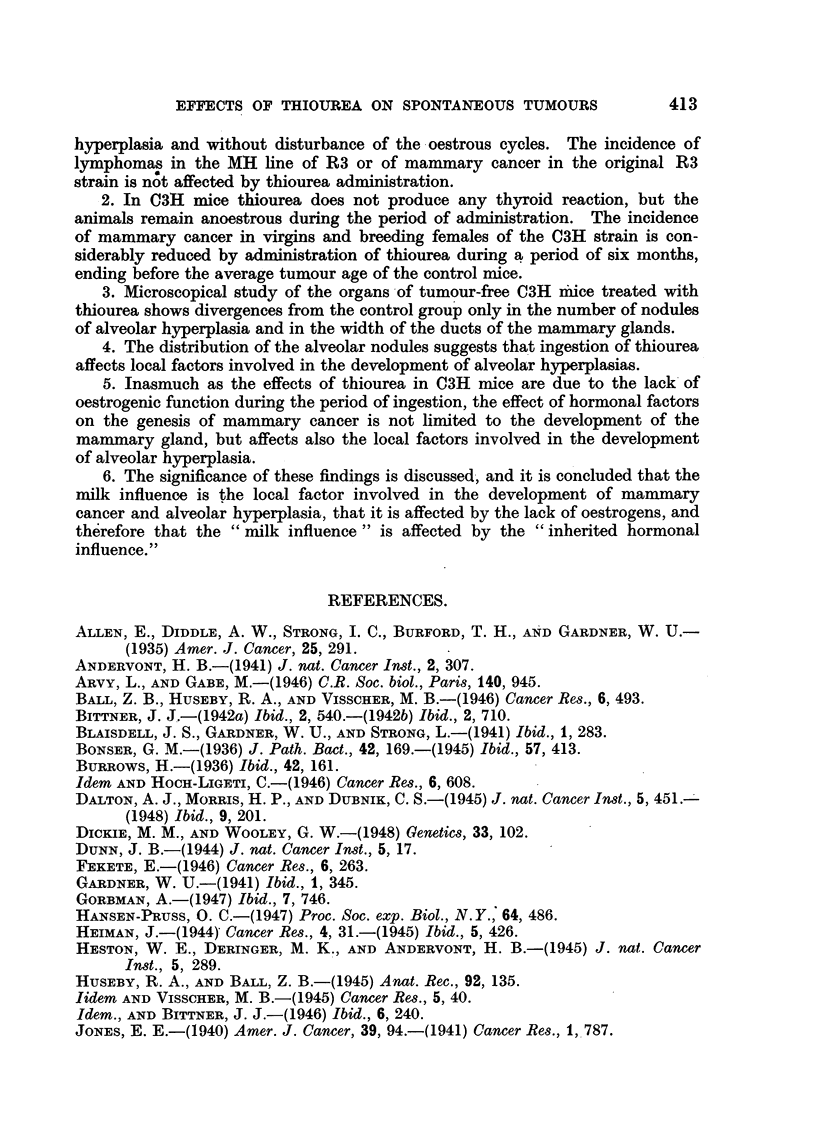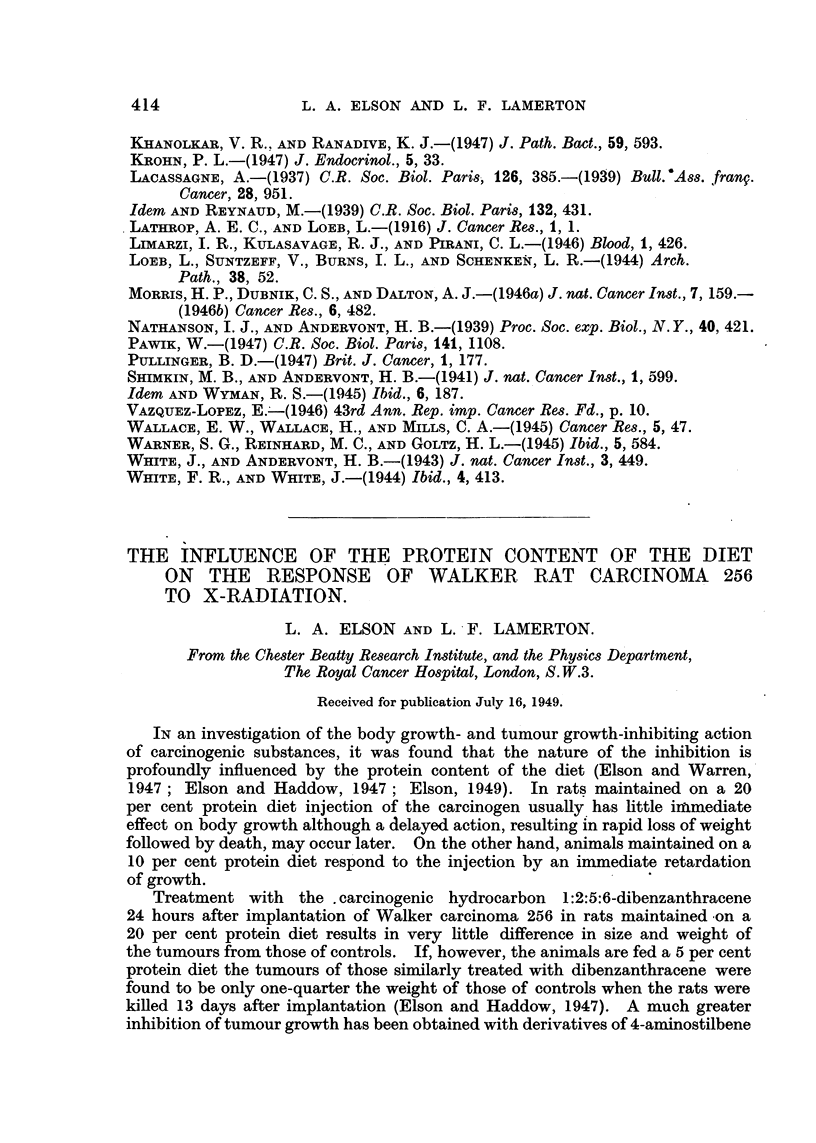# The Effects of Thiourea on the Development of Spontaneous Tumours in Mice

**DOI:** 10.1038/bjc.1949.45

**Published:** 1949-09

**Authors:** E. Vazquez-Lopez

## Abstract

**Images:**


### THE EFFECTS OF THIOUREA ON THE DEVELOPMENT OF SPONTANEOUS TUMOURS ON MICE.

E. VAZQUEZ-LOPEZ.

*From the Laboratories of the Imperial Cancer Research Fund, London, N.W.7.*

Received for publication June 17, 1949.

THE experiments described in this paper were intended to test whether the hypothyroidism provoked by prolonged administration of goitrogenic substances such as thiourea affected the development of spontaneous mammary tumours in mice of high cancer strains in a way comparable to that of restricted diets. No definite conclusions were obtained with regard to the influence of the thyroid gland, but the results showed a remarkable reduction of mammary cancer in one of the two strains used. Preliminary experiments (Vazquez-Lopez, 1946; Morris, Dubnik and Dalton, 1946*a*, *b*) showed that thiourea ingestion produced a continuous anoestrus due to ovarian atrophy and the consequent underdevelopment of the mammary glands. The effect of thiourea was therefore more or less the equivalent of ovariectomy. Inasmuch as this effect depended on continuous ingestion it was decided to withdraw the drug after a certain period, thus restoring ovarian function and allowing the mammary glands to reach a stage of development comparable to that of the control animals before the average time of tumour appearance. In that case the effects observed could not be attributed to the underdeveloped condition of the gland, but could be due only to the lack of oestrogenic activity during a limited period of the life of the mice.

### MATERIAL AND METHODS

C3H and R3 female mice bred in this laboratory by brother-sister mating for several years were used. The R3 mice belong to two different lines, only one of which had the high incidence of mammary cancer characteristic of this strain. The other line originated some time ago in this laboratory after mating two animals from different litters with the intention of improving breeding performance. After some generations it was found that the progeny of this pair had lost the characteristic high incidence of mammary cancer and become a low cancer line. Simultaneously with the loss of mammary cancer incidence this line developed a high incidence of lymphomas in breeding females, and thus provided the opportunity of testing the effect of thiourea in another kind of spontaneous tumour. The animals of this line (MH) used in the present experiment belonged to the 5th to 8th generations after the original cross, and most of them were born between June and September, 1946. Continuous brother-sister mating of the animals selected as descendants of the females with the earliest leukaemia development maintained the same incidence of lymphoma and mammary cancer till the 16th generation.

Breeding females only were used from the two lines of R3 mice, and both breeders and virgins from the C3H strain. The breeders were allowed to rear their first litters and, after weaning, the mothers were taken for the experiment. The litters of the C3H breeders provided the majority of the virgins employed. In all groups care was taken to divide animals of the same litters between the experimental and control groups.

The animals were housed in metal boxes, 6 to 8 in each, and fed rat cubes and water *ad lib.* Breeders and virgins of the C3H strain were separated, but the two lines of R3 breeders were mixed together.

Thiourea was administered in the drinking water, beginning with a 0·1 per cent solution and increasing the concentration as much as possible without endangering the survival of the animals. The approximate amount of fluid ingested was determined by dividing the total content of the water bottles by the number of animals in the box. All the animals were individually marked. The age of the breeders at the beginning of thiourea ingestion was between 3 and 4 months. The virgins were 3 months old, so that the mammary gland was fully developed before the action of the drug started.

The period of administration for some of the R3 animals was a year, so that most of the animals with tumours died during the period of ingestion. For the C3H mice, both breeders and virgins, it was 6 months, so that the animals were under normal conditions again when 9 or 10 months old. They were weighed regularly and inspected daily for the appearance of tumours. Vaginal smears were taken several times both during the period of thiourea ingestion and afterwards. In the virgins the presence of oestrus was also established before the start of the experiment to make sure that they had reached sexual maturity. The animals were allowed to live their full life span ; only those in moribund condition were killed.

The tumours, as well as ovaries, uterus and adrenal glands of mice in the control and experimental groups were fixed for histological study. The thyroid was also examined and whole mounts of the mammary glands prepared in the usual way. The most detailed study was made in the C3H virgins of both experimental and control groups.

### *Effects of Thiourea.*

### *General effects.*

There were very pronounced differences between the effects of thiourea in the two strains R3 and C3H. In both lines of R3 mice, those with high and low cancer incidence, the ingestion of the 0·1 per cent solution had no effect on the appearance, weight or water intake of the animals, and the strength of the solution could be increased to 0·5 per cent and maintained for long periods. Even concentrations of 1 per cent could be used before the mice showed toxic signs such as loss of weight and reduction of water intake. If the solutions were kept between 0·2 per cent and 0·5 per cent the R3 mice could be maintained for most of their lives without any apparent difference from the controls. The average length of the experiment was 10 months.

The C3H mice, both virgins and breeders, appeared on the contrary extremely susceptible to thiourea. Some animals died during the first days of ingestion, and even with a 0·1 per cent concentration they showed marked intolerance, mainly by loss of weight, restriction of fluid intake and poor appearance. The average weight of the animals during thiourea ingestion was 18 g. for the virgins and 21 g. for the breeders, compared with 24 and 28 g. for the control groups. The water intake was progressively reduced to a minimum of about 2 ml. per animal per day, and if the concentration of the drug was not reduced, many animals died. It was possible to reach a concentration of 0·5 per cent in most boxes, but it could be maintained only for one or two weeks. Solutions of 0·2 and 0·1 per cent were used most of the time, and even with these doses the intolerance to the drug and mounting mortality necessitated the withdrawal of the drug after 4 or 5 months. After some days without thiourea the mice recovered and the drug could then be administered again, but the recurrence of toxic symptoms was rapid. It was preferable to maintain the 6 months' period without interruption, even at the risk of losing more animals in the last months of treatment.

The doses administered were therefore variable for the different animals and for different periods for the same animal, but from the approximate estimate of water intake it may be assumed that the minimum of thiourea ingested per day was between 2 and 3 mg. for the C3H mice. For most R3 strain mice it was maintained at a level of about 15 mg. per day during most of the experiment, and during some periods it reached 30 mg. daily.

*Effects on the thyroid gland.*

In the R3 mice the ingestion of thiourea provoked the typical hypertrophy and hyperplasia with great vascularity, loss of colloid, etc. This effect was evident even with a 0·2 per cent solution, and became more pronounced in proportion to the length of treatment and increased concentration. As the majority of the animals with tumours died during the period of thiourea ingestion, the maximum effect was observed directly. The mice surviving after the withdrawal of the drug had thyroids of normal size and structure without any signs of the former hyperplasia.

The effects of thiourea in C3H mice were observed in the experimental animals that died during the period of ingestion of the drug. As the great majority of these mice restricted their water intake to a minimum in the weeks before their death, it could be argued that they were not actually under the action of the drug. For this reason additional mice not included in the experiment were used to test the effect under identical conditions, and were killed when the actual ingestion of thiourea was known.

In all the C3H mice of the experiment the results of the examination of the thyroid gland were similar. Thiourea administered in the drinking water did not produce hyperplasia or hypertrophy of the thyroid. There was clearly congestion of the organ which might cause a slight increase in size, but the histological picture remained normal, showing follicles filled with colloid and lined by cubic epithelium. None of the mice that died during the period of thiourea ingestion showed changes in the microscopical picture. Among 20 additional female adult mice killed after drinking solutions of 0·2 to 0·5 per cent for periods of a week to 2 months, with actual ingestion of about 5 mg. daily of thiourea, the size of the thyroid clearly increased in only one, in which also histologically the height of the cells and their disposition in solid acini without colloid corresponded to the typical picture of thyroid hyperplasia such as is found in R3 mice.

It is probable then that thiourea failed to produce any changes in the thyroid, but the possibility that some of the C3H mice reacted with hyperplasia cannot be excluded with certainty. Dalton, Morris and Dubnik (1945) and Morris *et al.* (1946*a*) found the typical hyperplasia and the expected increase in size in C3H mice fed thiourea for long periods. In a more recent paper (Dalton, Morris and Dubnik, 1948) they noted the wide individual variations in thyroid weight of thiouracil-treated mice. This lack of reaction to thiourea distinguished the C3H mice used in these experiments from the Andervont line used by Dalton *et al.* Strain differences in the thyroid reaction to thiouracil have been reported by Dickie and Wooley (1948).

*Effects on oestrous cycles and mammary gland.*

The effects of thiourea on the oestrous cycles differed sharply in the two strains used in these experiments. In the R3 mice both of the high and the low mammary cancer lines, the oestrous cycles remained unaffected even with the strong concentrations of thiourea which could be administered. Sometimes slight lengthening of the dioestrous intervals was observed during ingestion of 0·5 per cent and 1 per cent solutions. Most animals had normal oestrous cycles for the whole of the experiment.

C3H mice became anoestrous very soon after beginning thiourea administration and remained so during the whole time of ingestion. Smears taken at intervals during two week periods in each box were always negative. Morris *et al.* (1946*a*) found regular oestrus only in 3 mice among 21 fed thiourea. Krohn (1947), using stock albino mice injected daily with propyl-thiouracil, also found irregularities and complete disappearance of cycles. Gorbman (1947) observed an inhibitory action of thiourea on the gonads in mice. Arvy and Gabe (1946) and Pawick (1947) reported inhibition of the oestrus by thiourea and thiouracil in albino mice without any simultaneous hyperplastic action upon the thyroid after months of treatment.

Vaginal smears were also taken after discontinuing thiourea ingestion. Within a period of two weeks to a month the cycles were restored to normal. This was also the experience of Krohn (1947).

The mammary glands of the animals that died during the period of treatment showed changes correlated with the state of ovarian function. In R3 mice no difference from the controls was observed. In C3H mice absence of oestrogenic activity produced progressive atrophy with few ducts and stunted branching. Alveolar nodules were seen occasionally.

*Effects on tumour incidence.*

*Lymphomas.*—Under this title are included all the tumours of blood-forming organs, mainly lymphatic glands, thymus, spleen, etc., including the several types found in the strains with a high spontaneous incidence of such tumours.

The results of thiourea administration are given in Table I. No differences from the controls were found in the tumour incidence or in tumour age. The earlier average age of the experimental group was not significant, and the period of survival of non-tumour animals was slightly longer than in the controls. The incidence of other tumours was also the same in both groups, and that of mammary carcinoma showed the low figures (4 per cent) characteristic of the MH line of the R3 strain.

*Mammary cancer in R3 breeders.*—In the original R3 line with high cancer incidence the results given in Table II are similar in the thiourea-treated mice and in the controls. The small difference in favour of the thiourea group was not significant, for the small number of animals and the lower tumour age of this group was also against any inhibitory effect of thiourea. All the tumours appeared during the period of treatment when some of the mice had received thiourea for 10 months without interruption.

*Mammary cancer in C3H breeders.*—Of 49 mice at the start of the experiment, only 29 (effective number) reached the age at which the earliest tumour appeared. The other 20 died as a consequence of thiourea ingestion. Eight of the 29

TABLE I.—*Lymphoma Incidence in R3 Breeding Females (MH subline).*

| | Total number. | Effective number. | Mice with tumour. | %. | Average tumour age. | Average age of death in non-tumour mice. | Other tumours. |
|---|---|---|---|---|---|---|---|
| Controls | 51 | 42 | 19 | 46 | 384 days (216–701) | 431 days (229–730) | 2 mammary cancers. 2 hepatomas. |
| Thiourea | 55 | 52 | 22 | 42 | 321 days (146–621) | 454 days (253–591) | 2 mammary cancers. 1 hepatoma. |

TABLE II.—*Mammary Tumour Incidence in C3H and R3 Breeding Females.*

| | Total number. | Effective number. | Mice with tumour. | %. | Average tumour age. | Death age non-tumour animals. | Thyroid hyper-plasia. | Period of anoestrus. |
|---|---|---|---|---|---|---|---|---|
| C3H controls | 53 | 50 | 27 | 54 | 377 days (222–562) | 421 days (242–580) | — | — |
| C3H thiourea-treated | 49 | 29 | 8 | 28 | 457 days (367–538) | 514 days (383–677) | — | + |
| R3 controls | 7 | 7 | 7 | 100 | 435 days (301–592) | — | — | — |
| R3 thiourea-treated | 11 | 10 | 7 | 70 | 348 days (135–442) | 360 days (267–544) | + | — |

developed mammary cancer. No tumour appeared during the period of treatment. The earliest developed in a mouse 367 days old, 80 days after the withdrawal of thiourea. This was only 10 days less than the average tumour age for the control group, which was 377 days. These figures confirmed the assumption that ovarian function and mammary development were normal in the experimental animals at the average tumour age of the normal mice.

In spite of these normal conditions the percentage of mammary cancer in the thiourea-treated mice was 28 per cent compared with 54 per cent for the controls. The difference is statistically significant ($\chi^2 = 3{\cdot}96$). I am indebted to my colleague, Dr. Spicer, for the statistical analysis of this and the other results of the experiment. The average tumour age was increased in the experimental animals, and so too was the average life-span of the mice which did not develop tumours compared with those in the control group. In Table II are included the relevant figures of the breeders in the high mammary cancer lines, together with some details of the effects of thiourea on thyroid hyperplasia and oestrous cycles.

*Mammary cancer in C3H virgins.*—Twenty-one animals died during the period of thiourea ingestion, and 64 mice survived the 6 months of treatment and reached the period of appearance of the first tumour. Five among the 64 developed mammary cancer (8 per cent), at the average age of 571 days. In the control group 40 out of 96 animals (43 per cent) developed tumours at the average age of 432 days. The earliest tumour in the controls appeared in a mouse 254 days old. The animals treated were 270 days old when thiourea was withdrawn, so that ovarian activity and mammary gland development were presumably normal not only long before the average tumour age of the controls, but also very nearly when the first tumour appeared among the normal mice. Assuming that after the end of thiourea administration the mice needed a month for total recovery, they would be comparable with the normals when 300 days old. Only 4 mammary cancers developed in the control group below this age.

The decrease in cancer incidence in the thiourea-treated virgins was highly significant ($\chi^2 = 20{\cdot}9$), and confirms the results in C3H breeders. In this group the average tumour age was also later than in the controls. The average life span of mice without tumours was longer, but with smaller differences from the control mice than in the breeders.

### *Microscopical Study of Organs in Thiourea-Treated C3H Mice.*

A detailed study of the organs of C3H mice after longer periods of thiouracil and thiourea ingestion was published by Dalton *et al.* (1948). The animals were killed or died during treatment, and although the incidence of mammary cancer was reduced from 94 per cent to 17 per cent (Morris *et al.*, 1946*b*), the changes observed due to the continuous action of the drug may have masked those directly related to the inhibition of tumour development.

Huseby, Ball and Visscher (1945) also described alterations found in C3H mice fed restricted diets which completely inhibited mammary cancer, and Huseby and Ball (1945) those in A mice similarly treated. In both cases the restricted diets were maintained throughout the life of the animals and were effective at the time of examination. In the present experiments the withdrawal of thiourea after a limited period of administration allowed restoration of hormonal function, but the inhibition of tumours persisted. The changes observed in these circumstances should be more specifically related to the processes of cancer formation.

TABLE III.—*Mammary Cancer Incidence in C3H Virgins.*

| | Total number. | Effective number. | Mice with mammary cancers. | %. | Average tumour age. | Average death age in tumour mice. | Other tumours. |
|---|---|---|---|---|---|---|---|
| Controls | 96 | 94 | 40 | 43 | 432 days | 553 days | 4 lymphoma. |
| | | | | | (254–670) | (364–716) | 1 lung cancer.* |
| Thiourea treated | 85 | 64 | 5 | 8 | 571 days | 568 days | 4 lymphomas. |
| | | | | | (410–673) | (424–757) | 1 epithelioma. |

* Animal with mammary cancer also.

### EXPLANATION OF PLATE.

FIG. 1.—Mammary gland of thiourea-treated mouse. C3H virgin, 551 days old. (× 6.)
FIG. 2.—Mammary gland of control mouse of the same age. C3H virgin, 539 days old. (× 6.)
FIG. 3.—Mammary gland of thiourea-treated mouse showing great dilatation of ducts and terminal bulbs. C3H virgin, 749 days old. (× 6.)
FIG. 4.—Mammary gland of control mouse of the same age. C3H virgin, 716 days old. (× 6.)

Vazquez-Lopez.

Two groups of C3H virgins of similar ages were compared. Forty control mice between 459 and 716 days old (average 574 days) and 50 thiourea-treated, from 467 to 757 days old (average 574 days), were available for microscopical study. All were tumour-free. Tumour-bearing mice with and without thiourea treatment were compared separately, since histological changes in these mice might be secondary effects of the tumours. Ovaries, uterus, adrenal gland, thyroid and the complete set of mammary glands in each animal were studied.

*Mammary glands.*

In mice studied from 150 days onwards after the end of thiourea administration the mammary glands were identical to the glands of control mice of the same age in general structure, pattern, branching and extension. In both groups there was a progressive but very gradual decrease in size and richness of branching, and in most cases the development of the glands was correlated with the general condition and weight of the mouse rather than with its age. Fig. 1 and 2 show glands of control and experimental mice of approximately the same age.

The two main differences between thiourea-treated and control mice are the number of nodules of alveolar hyperplasia and the width of the ducts. The alveolar nodules of one gland or even of all the glands in a single mouse cannot be used to distinguish a thiourea-treated from a control mouse ; in some thiourea-treated animals the number and distribution of nodules is the same as in the controls. The group as a whole, however, shows significant differences, both in the number and in the distribution of nodules. The counts of the total number of nodules in tumour-free mice with and without thiourea are given in Table IV.

TABLE IV.—*Alveolar Nodules in C3H Mice (Virgin).*

| | Number of mice. | Average age. | Number of mice with nodules. | %. | Total of nodules. | Average number of nodules per mouse. |
|---|---|---|---|---|---|---|
| Controls | 40 | 574 days (456–716) | 30 | 75 | 526 | 17·3 |
| Thiourea-treated | 50 | 574 days (447–757) | 39 | 78 | 310 | 7·3 |

A small proportion of animals, about the same in both groups, had none. In those mice the glands were poorly developed and almost atrophic, and although the atrophy of the mamma should not prevent the persistence of the alveolar hyperplasia, it is most probable that the absence of nodules reflects the general condition of the animals rather than any specific cause. The thiourea-treated mice, as a whole, had considerably fewer nodules of alveolar hyperplasia than the control mice, and the average number of nodules per mouse was less than half the figure for the controls.

There are no records of comparative counts in similar experiments with reduced mammary cancer incidence, but Huseby and Bittner (1946) report that less than 1 nodule per 4 glands was found in some animals more than 340 days old kept on the restricted diet in the experiments of Huseby *et al.* (1945).

The distribution of the nodules per gland given in Table V shows additional points of interest. The smaller number in the thiourea-treated mice was not due to indiscriminate reduction affecting equally all the glands of each animal, but to a clear decrease of the number of glands with nodules. Only 32 per cent of the glands in the thiourea-treated mice had nodules compared with 51 per cent in the control group. There were also significant differences in the distribution and the average number of nodules for glands in each group, but the main effect of the thiourea administration is to increase the number of glands without any nodules. As the hormonal and systemic influences are the same for all the mammary glands in each mouse the cause of these differences between glands in the same mouse must be the result of local factors whose activity is restricted to the fields represented by particular glands. Thiourea acts systemically mainly by stopping ovarian function and oestrogen production. The effect of this lack of oestrogen, manifested in the different numbers of alveolar nodules, seems to depend on local factors independent of the general morphogenetic action.

TABLE V.—*Alevolar Nodules: Distribution per Gland.*

| | Number of glands. | Number of glands with nodules. | %. | Total number of nodules. | Average number of nodules per gland. |
|---|---|---|---|---|---|
| Controls | 370 | 190 | 51 | 526 | 2·7 |
| Thiourea-treated | 474 | 150 | 32 | 310 | 2 |

TABLE VI.—*Alveolar Nodules in C3H Mice (Virgins) with Tumours.*

| | Number of mice. | Number of mice with nodules. | %. | Total number of nodules. | Average number of nodules per mouse. |
|---|---|---|---|---|---|
| Controls | 18 | 18 | 100 | 462 | 25·7 |
| Thiourea-treated | 5 | 5 | 100 | 73 | 14·2 |

TABLE VII.—*Alveolar Nodules: Distribution per Gland.*

| | Number of glands | Number of glands with nodules. | %. | Total number of nodules. | Average number of nodules per gland. |
|---|---|---|---|---|---|
| Controls | 145 | 122 | 84 | 462 | 3·7 |
| Thiourea-treated | 43 | 36 | 83 | 73 | 2·0 |

The number of alveolar nodules in glands of control and thiourea-treated mice with tumours was also counted and the results are given in Table VI. There is a notable increase in the number compared with the tumour-free animals, but the difference between control and experimental groups persists. The distribution per gland is given in Table VII. The small number of animals makes the values of doubtful significance. The average number per gland in the thiourea-treated mice remains the same as in tumour-free animals. The percentage of glands with nodules is equal in both groups of mice without difference between thiourea-treated and controls.

The other difference between the mammary glands of the control and experimental mice affected the glandular ducts. It was not visible in all the animals, but usually, when it existed, affected all the ducts and glands of the animal to the same degree. It appeared in mice under 400 days old, and increased in frequency with age. It consisted in a considerable dilatation of the ducts, which reached three or four times the width of the normal. It is mostly visible in the main duct, but affected the whole extension of the gland, including the end bulbs and lateral buds, which appeared almost spherical. At the post-mortem examination the ducts appeared full of whitish material, which made them conspicuous to the naked eye. It seemed therefore to be due to engorgement by a secretory product, but it could exist in glands without any indication of alveolar proliferation. Fig. 3 shows glands of treated mice with wide dilated ducts, and Fig. 4 glands of control mice of the same age.

Dilatation of ducts has been described in oestrogen-treated animals of both sexes (Burrows, 1936 ; Gardner, 1941) and by Bonser (1936) without association with proliferating acini. In cross-suckling experiments, wide dilated ducts with blunt or rounded tips, similar to those found in thiourea-treated mice, have been found in oestradiol-treated Strong A males and C57 normal females, nursed by C3H mothers (Khanolkar and Ranadive, 1947).

*Other organs.*

It has been already mentioned that the thyroid gland of the experimental mice did not show any alteration in histological structure in mice which previously had suffered hyperplasia during thiourea administration. The ovaries and uterus of control and treated mice did not differ in any particular. Some ovaries appeared in regressed condition without corpora lutea or follicular development ; this occurred with the same frequency in both groups. Most ovaries had follicles in all stages of development, and some of the corpora lutea became hyalinized, forming large homogeneous masses with few cells. This formation of corpora albicantis is probably a strain characteristic, since Fekete (1946) found them in the dba high cancer strain, but never in the C57 black strain. Huseby *et al.* (1945) found them only in one C3H mouse with mammary cancer, and in none of the diet restricted animals of their experiment. That is probably another of the characteristics in which the C3H mice used here differ from those in American laboratories. The corpora albicantis were very prominent among the cancer-bearing animals, and their ovaries owed their large size partly to these structures. Apart from their size, however, the ovaries of mice with tumours showed mature follicles and abundant corpora lutea, which suggested normal activity. These findings were contrary to the results of Allen, Diddle, Strong, Burford and Gardner (1935) in several strains of mice, including the C3H.

In all groups the condition of the uterus reflects that of the ovaries having tall epithelium, extensive glandular development and loose cellular stroma in the animals with well functioning ovaries and signs of regression in those with ovarian atrophy.

No differences were found in the adrenal glands of thiourea-treated mice compared with the controls. The most important change, affecting both groups equally, was the presence of amyloid degeneration, which appeared on the borderline between cortex and medulla and progressed towards the glomerular zone with advancing age. In the oldest animals amyloid invaded most of the cortex, and only two or three layers of cortical cells remained intact. The medulla was never affected. " Brown degeneration " was, on the contrary, never found in C3H mice, but was almost constant in R3 animals of high or low mammary cancer lines. Amyloid degeneration of the same type was described by Blaisdell,

Gardner and Strong (1941) in C3H mice and in other strains with and without cancer susceptibility. Dunn (1944) found it also in C3H mice and their hybrids.

The incidence and extension of adrenal amyloidosis was independent of thiourea treatment, but a difference was found between the tumour and non-tumour-bearing animals of each group, at the same age. Table VIII gives the results in non-tumour animals, 92 per cent of which showed variable degrees of amyloid invasion. Table IX shows the frequency of amyloid in animals with mammary cancer and some other spontaneous tumours. The mammary cancer group includes control and thiourea-treated mice. The percentage of tumour-bearing animals with amyloid was 41 per cent, and the difference from tumour-free mice of similar age is statistically significant ($\chi^2 = 17{\cdot}4$). It cannot be decided if this resistance to adrenal degeneration is directly related to the tumour development. The fact that tumour-bearing animals maintain better ovarian structure than non-tumour animals of the same age seems to favour instead a correlation between ovarian activity and adreno-cortical condition.

TABLE VIII.—*Amyloid Degeneration of Adrenals in C3H Virgins without Tumours.*

| | Number of mice. | Age. | Number with amyloid. | %. |
|---|---|---|---|---|
| Controls | 27 | 471–716 days | 25 | .. |
| Thiourea-treated | 25 | 525–749 „ | 23 | .. |
| Total | 52 | .. | 48 | 92 |

TABLE IX.—*Amyloid Degeneration of Adrenals in C3H Virgins with Tumours.*

| | Number of mice. | Age. | Number with amyloid. | %. |
|---|---|---|---|---|
| Mammary cancers | 16 | 423–715 days | 8 | .. |
| Lymphoma | 5 | 502–692 „ | 2 | .. |
| Epithelioma | 1 | 590 days | .. | .. |
| Total | 22 | .. | 10 | 41 |

### DISCUSSION.

From the observations described it may be concluded that the main effect of thiourea in C3H mice is the maintenance of anoestrus during the period of administration. The possibility of a temporary hypothyroidism cannot be excluded, but the significance of thyroid function for tumour production is very much reduced when it is considered that in R3 mice the incidences of mammary cancer and lymphoma are completely unaffected, in spite of considerable enlargement of the gland. If hypertrophy is accompanied by changes in the metabolic rate comparable to those taking place in rats or man, the influence of caloric intake *per se* is not very important in the genesis of malignant tumours. The possibility of a specific activity of thiourea on blood-forming tissues and their neoplasms suggested by the agranulocytic reactions in patients treated with goitrogenic substances is not supported by the results in the MH high lymphoma line which agree with clinical reports of treatment of leukaemic patients (Limarzi, Kulasavage and Pirani, 1946 ; Hansen-Pruss, 1947).

That lack of oestrogen is the effective factor is shown also by the normal incidence of cancer corresponding with regular oestrus in spite of thiourea administration in R3 mice. The correlation between ovarian inhibition and decrease in mammary cancer proved by Lathrop and Loeb in 1916 is one of the oldest and probably the most often quoted experiment in hormonal cancer research. The results of ovariectomy are confirmed by the effects of hormones which antagonize oestrogenic activity such as that of testosterone (Lacassagne, 1939; Nathanson and Andervont, 1939; Lacassagne and Reynaud, 1939; Jones, 1941; Heiman, 1944). The action of progesterone is still a matter for discussion (Lacassagne, 1937; Heiman, 1945; Burrows and Hoch-Ligeti, 1946). Indirect ways of decreasing oestrogenic activity give, however, less clear results. Although low cystine diets completely inhibit mammary cancer in C3H mice (White and Andervont, 1943), the effects of stilboestrol pellets which restore the development of the mammary glands raise the incidence only to 44 per cent compared with 92 per cent in the controls (White and White, 1944). In similar experiments, Ball, Huseby and Visscher (1946) found that in spite of continuous administration of stilboestrol the mammary glands of Strain A mice maintained on restricted diets during 15 months were considerably less developed than those of control mice. The possibility of other hormonal factors influencing mammary cancer development is also suggested by the results of Shimkin and Wyman (1945), who found greater reduction in adrenalectomized C3H mice than after ovariectomy, and by the observation of Wallace, Wallace and Mills (1945) of persistence of effects of temperature in mammary cancer incidence after ovariectomy.

The results of temporary lack of oestrogenic activity caused by thiourea show that reduction of mammary cancer is not due to defective glandular development, and therefore that the hormonal effects are not limited to the morphogenesis of tissue susceptible to carcinogenic agents. Mammary glands of two groups of mice of the same strain with different cancer incidences have identical morphology, and only differ in the total number of nodules of alveolar hyperplasia in each group. The significance of these nodules of alveolar hyperplasia in relation to carcinogenesis has been indicated by several authors. The same factors involved in the genesis of mammary cancer are necessary for the alveolar hyperplasia. Huseby and Bittner (1946) have shown by the study of C3H and hybrid mice with or without the " milk influence " that this influence is necessary for the presence of nodules. The importance of the "inherited hormonal influence" is shown by the absence of nodules in virgins of the A strain. The significance of each factor for the development and persistence of the alveolar hyperplasia has been studied by Pullinger (1947), who provoked early appearance of nodules in males and ovariectomized virgins of R3 and C3H strains by injection of measured doses of oestrogens. Although alveolar hyperplasia is induced by oestrogens in mice without the milk influence, the persistence and autonomy of the alveolar hyperplasia requires the presence of the Bittner factor, and they disappear gradually in mice deprived of it. There is no nodular formation in A virgins injected with the amounts of oestrogens that are effective in other strains.

These are sufficient reasons for considering the alveolar hyperplasia as the result of the sum of hormonal and milk-transmitted influences, affecting the mammary glands of strains genetically susceptible to spontaneous tumour formation. The number of glands with nodules is considerably smaller in thiourea-treated mice. The hormonal influences in the thiourea treated mice seem to have been sufficient to reach the level necessary for the development of nodules in the same proportion of mice as in the controls, in spite of the temporary inhibition of oestrus. The distension of ducts found also in the thiourea-treated group seems to be a sign of excessive oestrogenic stimulation, and may indicate compensatory excess after thiourea withdrawal.

If the hormonal influences are sufficient for the alveolar development the smaller number of glands with nodules, which is the main result of thiourea administration, must be due to local factors. The lack of oestrogens (and its possible compensation afterwards) affects all the glands of the mice equally. These results point to the possibility of effects on local factors and producing different consequences in each gland of the same animal. The local factor involved in the persistence of alveolar nodules is the milk factor, and the distribution of nodules suggests that it is affected by thiourea administration.

There are no grounds for supposing that thiourea can directly influence the milk factor, but there are some in favour of an indirect action resulting from the lack of oestrogens. Apart from the considerable number of experiments on the effects of oestrogen administration on cancer incidence, there is evidence even in normal circumstances of hormonal influences in breeding and forced breeding, increasing the number of tumours. The number of litters also increases the incidence in breeders (Jones, 1940 ; Andervont, 1941). The higher incidence in mice born in later litters compared with the earliest from the same mother has been explained by the different concentration of the milk influence with advancing age (Bittner, 1942*a*). If the increased hormonal stimulation is the cause of increase in the amount or activity of the milk influence, it is probable that the converse holds true also, and that the lack of hormones acts in the opposite sense.

The relative importance of the hormonal influence among the three factors necessary for the genesis of spontaneous mammary cancer in mice is not exactly known. Opinions range from that of Shimkin and Andervont (1941), who consider the hormonal factors as necessary only for the creation of a substratum for the carcinogenic action of the factor transmitted by the milk, to that of Warner, Reinhard and Goltz (1945), who maintain the dominance in certain cases of the inherited susceptibility upon the concentration of the milk agent. Loeb, Suntzeff, Burns and Schenken (1944) believe that the oestrogens act by stimulating the growth of the mammary tissue and sensitizing it to all types of stimuli, and Bonser (1945) as a developing factor acting upon breast tissues already sensitized by the milk factor. The only common feature of all these opinions is to consider the different factors as completely independent variables. On this basis the opinion of Bittner (1942*b*) that each influence must be considered as complementary to the others and all equally important is, besides being non-controversial, nearest to the observed facts. But if there are some factors which are affected and increased, or even created *de novo* by interaction of the others, the relative significance of each would not be the same.

The effect of genetic susceptibility on some aspects of the milk factor activities has been already proved by Heston, Deringer and Andervont (1945). The effect of the inherited hormonal influence as suggested by the result of the inhibition of oestrogenic activity would be another example of similar interaction.

### SUMMARY.

1. The effects of thiourea in mice depend on strain characteristics. R3 mice, whether of high or low mammary cancer sublines, react with great thyroid hyperplasia and without disturbance of the oestrous cycles. The incidence of lymphomas in the MH line of R3 or of mammary cancer in the original R3 strain is not affected by thiourea administration.

2. In C3H mice thiourea does not produce any thyroid reaction, but the animals remain anoestrous during the period of administration. The incidence of mammary cancer in virgins and breeding females of the C3H strain is considerably reduced by administration of thiourea during a period of six months, ending before the average tumour age of the control mice.

3. Microscopical study of the organs of tumour-free C3H mice treated with thiourea shows divergences from the control group only in the number of nodules of alveolar hyperplasia and in the width of the ducts of the mammary glands.

4. The distribution of the alveolar nodules suggests that ingestion of thiourea affects local factors involved in the development of alveolar hyperplasias.

5. Inasmuch as the effects of thiourea in C3H mice are due to the lack of oestrogenic function during the period of ingestion, the effect of hormonal factors on the genesis of mammary cancer is not limited to the development of the mammary gland, but affects also the local factors involved in the development of alveolar hyperplasia.

6. The significance of these findings is discussed, and it is concluded that the milk influence is the local factor involved in the development of mammary cancer and alveolar hyperplasia, that it is affected by the lack of oestrogens, and therefore that the " milk influence " is affected by the " inherited hormonal influence."

### REFERENCES.

ALLEN, E., DIDDLE, A. W., STRONG, I. C., BURFORD, T. H., AND GARDNER, W. U.—(1935) *Amer. J. Cancer*, **25**, 291.
ANDERVONT, H. B.—(1941) *J. nat. Cancer Inst.*, **2**, 307.
ARVY, L., AND GABE, M.—(1946) *C.R. Soc. biol., Paris*, **140**, 945.
BALL, Z. B., HUSEBY, R. A., AND VISSCHER, M. B.—(1946) *Cancer Res.*, **6**, 493.
BITTNER, J. J.—(1942*a*) *Ibid.*, **2**, 540.—(1942*b*) *Ibid.*, **2**, 710.
BLAISDELL, J. S., GARDNER, W. U., AND STRONG, L.—(1941) *Ibid.*, **1**, 283.
BONSER, G. M.—(1936) *J. Path. Bact.*, **42**, 169.—(1945) *Ibid.*, **57**, 413.
BURROWS, H.—(1936) *Ibid.*, **42**, 161.
*Idem* AND HOCH-LIGETI, C.—(1946) *Cancer Res.*, **6**, 608.
DALTON, A. J., MORRIS, H. P., AND DUBNIK, C. S.—(1945) *J. nat. Cancer Inst.*, **5**, 451.—(1948) *Ibid.*, **9**, 201.
DICKIE, M. M., AND WOOLEY, G. W.—(1948) *Genetics*, **33**, 102.
DUNN, J. B.—(1944) *J. nat. Cancer Inst.*, **5**, 17.
FEKETE, E.—(1946) *Cancer Res.*, **6**, 263.
GARDNER, W. U.—(1941) *Ibid.*, **1**, 345.
GORBMAN, A.—(1947) *Ibid.*, **7**, 746.
HANSEN-PRUSS, O. C.—(1947) *Proc. Soc. exp. Biol., N.Y.*, **64**, 486.
HEIMAN, J.—(1944) *Cancer Res.*, **4**, 31.—(1945) *Ibid.*, **5**, 426.
HESTON, W. E., DERINGER, M. K., AND ANDERVONT, H. B.—(1945) *J. nat. Cancer Inst.*, **5**, 289.
HUSEBY, R. A., AND BALL, Z. B.—(1945) *Anat. Rec.*, **92**, 135.
*Iidem* AND VISSCHER, M. B.—(1945) *Cancer Res.*, **5**, 40.
*Idem.*, AND BITTNER, J. J.—(1946) *Ibid.*, **6**, 240.
JONES, E. E.—(1940) *Amer. J. Cancer*, **39**, 94.—(1941) *Cancer Res.*, **1**, 787.

KHANOLKAR, V. R., AND RANADIVE, K. J.—(1947) *J. Path. Bact.*, **59**, 593.
KROHN, P. L.—(1947) *J. Endocrinol.*, **5**, 33.
LACASSAGNE, A.—(1937) *C.R. Soc. Biol. Paris*, **126**, 385.—(1939) *Bull. Ass. franç. Cancer*, **28**, 951.
*Idem* AND REYNAUD, M.—(1939) *C.R. Soc. Biol. Paris*, **132**, 431.
LATHROP, A. E. C., AND LOEB, L.—(1916) *J. Cancer Res.*, **1**, 1.
LIMARZI, I. R., KULASAVAGE, R. J., AND PIRANI, C. L.—(1946) *Blood*, **1**, 426.
LOEB, L., SUNTZEFF, V., BURNS, I. L., AND SCHENKEN, L. R.—(1944) *Arch. Path.*, **38**, 52.
MORRIS, H. P., DUBNIK, C. S., AND DALTON, A. J.—(1946*a*) *J. nat. Cancer Inst.*, **7**, 159.—(1946*b*) *Cancer Res.*, **6**, 482.
NATHANSON, I. J., AND ANDERVONT, H. B.—(1939) *Proc. Soc. exp. Biol., N.Y.*, **40**, 421.
PAWIK, W.—(1947) *C.R. Soc. Biol. Paris*, **141**, 1108.
PULLINGER, B. D.—(1947) *Brit. J. Cancer*, **1**, 177.
SHIMKIN, M. B., AND ANDERVONT, H. B.—(1941) *J. nat. Cancer Inst.*, **1**, 599.
*Idem* AND WYMAN, R. S.—(1945) *Ibid.*, **6**, 187.
VAZQUEZ-LOPEZ, E.—(1946) 43*rd Ann. Rep. imp. Cancer Res. Fd.*, p. 10.
WALLACE, E. W., WALLACE, H., AND MILLS, C. A.—(1945) *Cancer Res.*, **5**, 47.
WARNER, S. G., REINHARD, M. C., AND GOLTZ, H. L.—(1945) *Ibid.*, **5**, 584.
WHITE, J., AND ANDERVONT, H. B.—(1943) *J. nat. Cancer Inst.*, **3**, 449.
WHITE, F. R., AND WHITE, J.—(1944) *Ibid.*, **4**, 413.

---

### THE INFLUENCE OF THE PROTEIN CONTENT OF THE DIET ON THE RESPONSE OF WALKER RAT CARCINOMA 256 TO X-RADIATION.

L. A. ELSON AND L. F. LAMERTON.